# Rapid birth-death evolution and positive selection in detoxification-type glutathione S-transferases in mammals

**DOI:** 10.1371/journal.pone.0209336

**Published:** 2018-12-26

**Authors:** Hui Ming Tan, Wai Yee Low

**Affiliations:** 1 Center for Bioinformatics, Perdana University School of Data Science, Serdang, Selangor, Malaysia; 2 The Davies Research Centre, School of Animal and Veterinary Sciences, University of Adelaide, Roseworthy, SA, Australia; California State University Fullerton, UNITED STATES

## Abstract

Glutathione S-Transferases (GSTs) are phase II detoxification enzymes that may have evolved in response to changes of environmental substrates. GST genes formed a multigene family and in mammals, there are six classes known as Alpha, Mu, Omega, Pi, Theta, and Zeta. Recent studies in phase I detoxification system specifically the cytochrome P450s provided a general explanation on why genes from a common origin such as those in a multigene family have both phylogenetically stable and unstable genes. Genes that participate in core functions of organisms such as development and physiology are stable whereas genes that play a role in detoxification are unstable and evolve in a process known as birth-death evolution, which is characterised by frequent gene gains and losses. The generality of the birth-death model at explaining the evolution of detoxification enzymes beyond the phase I enzyme has not been comprehensively explored. This work utilized 383 *Gst* genes and 300 pseudogenes across 22 mammalian species to study gene gains and losses. GSTs vary greatly in their phylogenetic stability despite their overall sequence similarity. Stable *Gst* genes from Omega and Zeta classes do not show fluctuation in gene numbers from human to opossum. These genes play a role in biosynthesis related functions. Unstable genes that include Alpha, Mu, Pi and Theta undergo frequent gene gain and loss in a process known as birth-death evolution. Gene members of these four classes are well known for their roles in detoxification. Our positive selection screen identified five positively selected sites in mouse GSTA3. Previous studies showed two of these sites (108H and 208E) were biochemically tested as important residues that conferred catalytic activity against the toxic aflatoxin B_1_-8,9-epoxide. The functional significance against aflatoxin of the remaining three positively selected sites warrant further investigation.

## Background

Genes that adopt a particular canonical protein fold and share amino acid sequence similarity above a certain threshold are grouped into a multigene family [1]. These genes have a common origin and through gene duplications and losses, a single ancestral gene can now be found as multiple copies spread throughout the genome. Some gene members of a multigene family have a phylogenetically stable gene copy number whereas others display a much more unstable gene copy number even over only short evolutionary distances since species divergence. The definition of ‘phylogenetic instability’ may be loosely defined but studies on stable versus unstable genes have relied on a sharp distinction between gene classes from the same multigene family that showed very different gene copy number variation when analysed on a specific evolution timeframe [2]. The term birth-death evolution describes unstable genes that undergo frequent duplications and loses. Presumably genes in rapid birth-death evolution have more opportunities for neofunctionalization, subfunctionalization and pseudogenization [3].

Until recently, there is no general explanation for why genes from a common origin such as those in a multigene family show differing patterns of gene gains and losses. In a study by James Thomas in 2007, he took advantage of the interesting evolution in the cytochrome P450 family, which is known to have gene members that play a role in detoxification and others with different roles such as in biosynthetic pathways, to explain stable versus unstable gene evolution [4]. It was found that stable genes participate in core functions of organisms such as development and physiology whereas unstable genes with rapid birth-death evolution mode play a role in adaptation to specific niches that require dealing with environmental toxins and pathogens. This finding was further supported by another study that extended the study on the evolution of the P450s to include both the vertebrate and invertebrates [5]. This simple model that has the power to explain not only the evolution of the cytochrome P450s family but also many of the multigene families that play a role in detoxification.

The detoxification system can be classified into three distinct phases that are commonly referred to as Phase I, II, and III, which act in an integrated manner [1]. Phase I and II involve chemical catalysis of lipophilic, non-polar compounds into a more water-soluble and less toxic form of chemicals. In Phase I, the cytochrome P450 monooxygenases (P450s) are involved and the general reaction that they perform are oxidation [6]. The oxidized product is passed on to Phase II enzymes, which performs reactions such as conjugation catalysed by GSTs, UDP-glucuronosyl transferases (UGTs), NATs [7] and sulfotransferases (SULTs) [8]. In Phase III, transporter proteins that belong to a family of proteins called the ABC transporters (for ATP-Binding Cassette) move conjugates formed from Phase II enzymes out of the cells.

GSTs is a multigene family that catalyse the formation of conjugates between glutathione (GSH) with various types of xenobiotic substrates. In humans, there are 16 cytosolic GSTs genes and they are made up of six classes, which are Alpha, Mu, Omega, Pi, Theta and Zeta [9]. In general, GSTs that are grouped within a class share greater than 60% identity whereas those with less than 30% identity are categorized into separate classes [1]. However, classification of GSTs was not based only on sequence alignments but also immunological relationships, substrate kinetic properties and protein structure comparisons. Similar to the P450s [2], GSTs can be categorised as biosynthetic- or the detoxification-type by mining the literature on known functions of these proteins, which are usually derived from biochemical studies [1]. To explore the generality of the model put forward by previous studies in explaining the patterns of gene gains and losses beyond just the phase I cytochrome P450s, we examined in detail the evolution of phase II Glutathione S-transferases and restrict the choice of species to include only 22 mammals with genomes assembled at the chromosome level [4, 5]. One main objective is to test the hypothesis of higher rate of gene gains and losses in detoxification- versus biosynthesis-type of GSTs. Another aim is to identify potentially undiscovered positively selected sites in mammalian GSTs, which can inform on functional changes of the enzymes [10]. Our choice of mammalian GSTs take into account the need for using only well assembled genomes to perform manual gene annotation since finding on gene gains and loses depends on accurate gene models, which in turn requires better draft assemblies. Performing our study on mammals can help us understand recent evolutionary changes that have shaped the human GSTs divergence.

## Materials and methods

### Human GST genes as a reference

There are 16 bona fide cytosolic human GST genes that are categorised into six classes, which are Alpha, Mu, Omega, Pi, Theta and Zeta [9, 11]. The DNA and protein sequences of these human GSTs were downloaded from the NCBI and their accession numbers are given in S1 Table [12].

### Manual annotations

Searches for suitable mammalian genomes to mine for GSTs began with a filter for “Mammalia” under the Organism category in the NCBI database in Jun 2015. Only species with assembly status at the chromosome level and have RefSeq assemblies were chosen. Altogether 23 species passed the filters and were used for further analyses. In order to find homologous GST genes, each of the 16 human reference GSTs was used as input for TBLASTN searches against 23 mammalian species with e-value cut-off of 1e^-5^. The overlapping coordinates of TBLASTN hits were merged and checked against the Genbank gene model annotations. Manual annotations were done with the Artemis software v16.0.0 where necessary [13]. In cases where more than one protein isoforms were found, the isoform was selected based on the highest BLASTP identity to human GSTs and its protein length was more than 200 amino acids. The cut-off protein length was chosen on the basis that average GST length of well annotated human, mouse and rat GSTs is 220 amino acids. For some of the genes, it was necessary to mine suitable transcripts from the NCBI database and then mapped back onto the gene model to best determine the accurate gene models.

Pseudogenes were identified by inactivating mutations that included stop codons, gene truncations and retrotransposed genes that are usually “dead on arrival” immediately following retrotransposition events [14, 15]. The term “partial” gene was used to denote genes that contained sequence gaps such as a gene broken into multiple contigs and its protein length was less than 200 amino acids. As the gene models in the NCBI contained errors and may have missing genes, we curated the annotations to ensure appropriate gene models were used for downstream analysis. URLs for curated sequences can be found here: https://bit.ly/2O5HPeb, https://bit.ly/2oG2tCl

### Phylogenetic tree and identification of orthologs

To study the phylogenetic relationships of GSTs, a maximum likelihood tree was constructed using all the 22 mammalian species GSTs. Altogether, 383 protein sequences were aligned by ClustalW and used as an input for tree construction in Mega v6 [16]. The human GSTS was used as an outgroup. A 100-replicate bootstrapped tree was used to assess the reliability of the phylogenetic tree. Jones-Taylor-Thornton (JTT) model was chosen for amino acid substituition. Nodes with less than 70% bootstrap support were collapsed and the resulting clades formed the basis of assignments of orthologous relationships. In cases where the bootstrap support were weak to group genes as orthologs, the gene order of GSTs including flanking non-GST genes were used to decide orthologous relationships. The final tree was drawn in figtree with collapsed nodes to clearly show the six GST classes [17].

### Rates of gene gain and loss in detoxification- and biosynthesis-type of GSTs

Following a literature survey on known GST functions from all classes found in mammals, it is possible to generally categorise each class as detoxification- or biosynthesis-type (S2 Table). Briefly, the procedure to functionally categorise a GST class started with PubMed searches for the term “GST function”. The corpus was then scanned for review studies that summarised GST functions and relevant cited papers from the reviews were followed. Given that GSTs were known as promiscuous enzymes, there were cases where it was difficult to place a GST class as strictly involved in detoxification or biosynthesis. For example, rat GSTO1-1 is known for its dehydroascorbate reductase activity, which is an important enzyme in the recycling of ascorbate (vitamin C) (i.e. a biosynthesis related function) [18]. However, the same enzyme may play a role in arsenic biotransformation due to its ability to reduce monomethylarsonic acid but further experiments have not supported it as a major player in this pathway [19, 20]. Therefore, it is more plausible that in evolution, the main function of GST Omega class is in biosynthesis rather than detoxification.

To infer gene gain and loss of mammalian GSTs across the 22 species phylogeny, three models in the DupliPHY-ML software, Pasimony, Birth—Death Innovation (BDI) and Birth—Death Innovation and Extinction (BDIE) were used [21]. Figtree v1.4.3 software was used to view phylogenetic tree that has node labels to indicate inferred gene number for each node. By using the 22 mammalian species phylogenetic tree the inferred gene gains and losses for each branch were calculated using a custom R script. The species tree was obtained from TimeTree [22].

### A screen for positive selection

Twenty-one sets of GSTs were analysed with CODEML v3.14 program in the PAML package [23]. This software was used to measure selective pressure by estimates of the rate ratio (ω) of non-synonymous (amino acid changes) to synonymous (silent changes) substitutions (dN/dS). ω < 1 is a sign of negative selection, which means the mutation is deleterious whereas ω = 1 means the sequence evolution is consistent with neutral evolution. If ω > 1, this indicates positive selection but detection of ω > 1 when averaged across all amino acid sites and lineages is rare since positive selection may not act on all sites. The site models, namely M7 and M8, allow ω to vary among sites and hence provide more powerful models to detect sign of positive selection. As implied by the name of the tool, it uses a maximum likelihood method to estimate parameters of models based on observed data (i.e. a gene tree and alignment of codons) [24]. Where possible, amino acid sequences of orthologous set were aligned using ClustalX2 v2.1 and then the nucleotide coordinates were mapped to the corresponding amino acid alignment using the program PAL2NAL [25]. In cases with unclear orthologous relationships, paralogs were also used in the gene set. Gaps were removed prior to running CODEML. The tree topology supplied for CODEML followed the maximum likelihood gene tree.

In Model 7, a β distribution is given 10 site classes of ω between 0 and 1. In Model 8, a class of sites that has ω > 1 is added to Model 7. Therefore, Model 8 is a positive selection model whereas Model 7 is its corresponding neutral model. Significance of positively selected sites found were evaluated using the likelihood ratio test (LRT) [26], which is a simple calculation of the test statistic *t*_*LR*_ = 2[*l*(*Model* 8)−*l*(*Model* 7)]. The P-value of the test statistic was evaluated with degree of freedom equals 2. To control for false positive results, we applied False Discovery Rate (FDR) test to adjust P-values for the 21 gene sets used for PAML so that FDR < 0.05 [27]. The range of the total tree length (S) and the number of taxa (T) should allow for relatively high power and accuracy in detection of positive selection (0.22 < S < 7.16, 6 < T < 31; [28]). The positively selected sites found were compared with those already known from the literature (S3 Table).

## Results

### Mammalian GSTs dataset and phylogetic tree

There were 23 mammalian species chosen based on assembly at the chromosome level because these assemblies were likely more complete when compared to other genomes that were only assembled at the contig or scaffold level (S4 Table). However, the assembly contig N50 in the platypus genome was only 11.6 kb and there were more than 200,000 scaffolds, which made this genome a highly fragmented one. An attempt to annotate this genome for GSTs only showed one complete GST found whereas all the other genes were broken into shorter contigs; the platypus genome was removed from further analysis.

**Fig 1:**
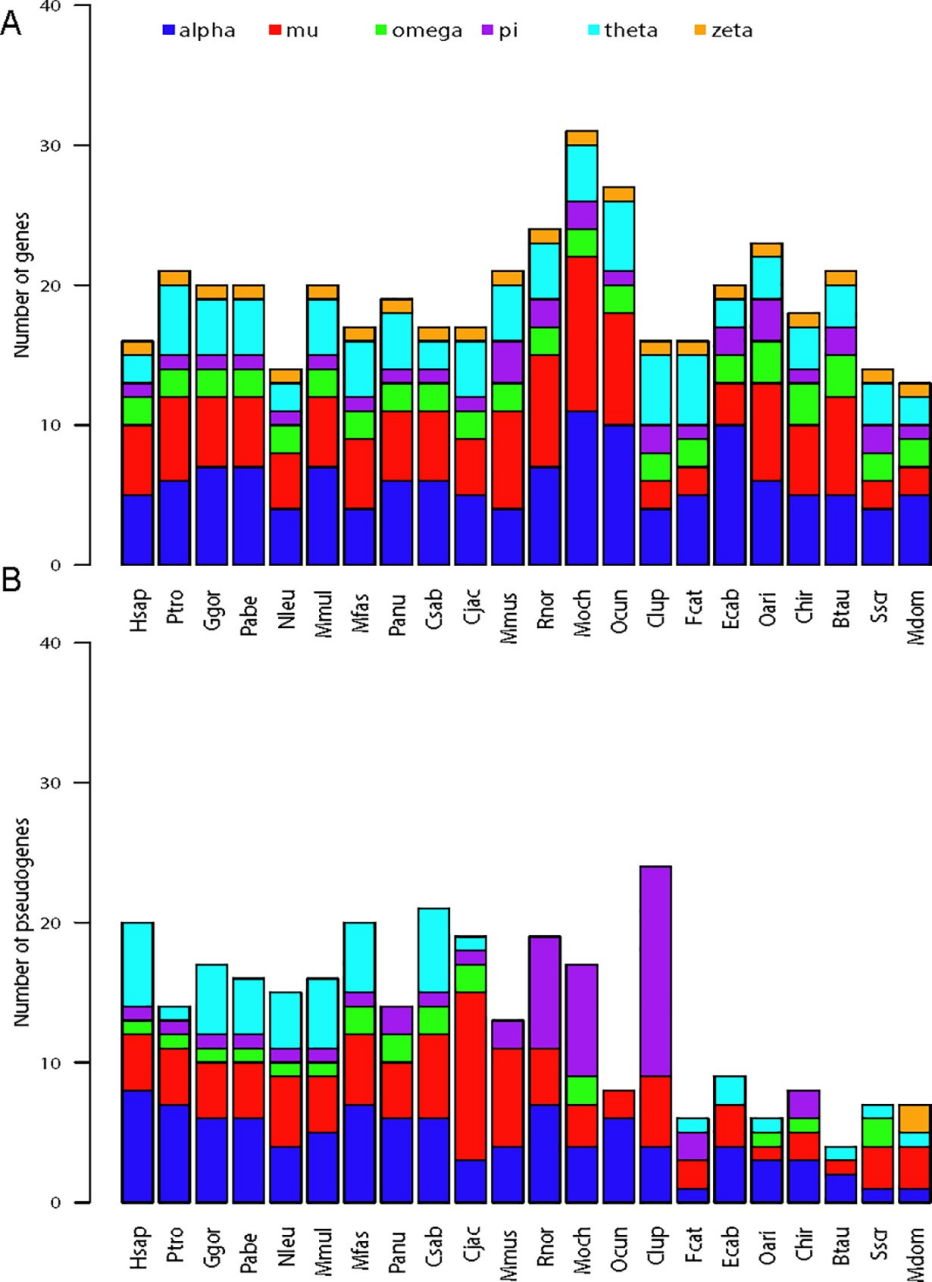
**Barplots showing distribution of number of (A) GST genes and (B) pseudogenes across the 22 mammalian species. For gene count, the number of partial genes was also included.** The species abbreviation is as follow: Hsap—*Homo sapiens*; Ptro—*Pan troglodytes*; Ggor—*Gorilla gorilla gorilla*; Pabe—*Pongo abelii*; Nleu—*Nomascus leucogenys*; Mmul—*Macaca mulatta*; Mfas—*Macaca fascicularis*; Panu—*Papio anubis*; Csab—*Chlorocebus sabaeus*; Cjac—*Callithrix jacchus*; Mmus—*Mus musculus*; Rnor—*Rattus norvegicus*; Moch—*Microtus ochrogaster*; Ocun—*Oryctolagus cuniculus*; Clup—*Canis lupus familiaris*; Fcat—*Felis catus*; Ecab—*Equus caballus*; Oari—*Ovis aries*; Chir—*Capra hircus*; Btau—*Bos taurus*; Sscr—*Sus scrofa*; Mdom—*Monodelphis domestica*.

Our search in 22 mammalian species identified 383 genes and 300 pseudogenes in the cytosolic GSTs multigene family (S1 Table). There were 42 “partial” genes because these genes may be broken up into more than one contig or they were found at contig edges. As the GSTs annotations in the NCBI contained errors, we curated the annotations and found 31 pseudogenes incorrectly annotated as genes, corrected the gene models of 26 genes and found one new goat gene. Altogether, there were 58 curated genes that differed from the NCBI annotations. All six classes of GSTs (Alpha, Mu, Omega, Pi, Theta, Zeta) were successfully identified in all species (Fig 1). The variation in GST gene number across the 22 species ranged from 14 to 31 and interestingly, the number of Zeta GST was conserved as a single copy gene from human to opossum. The total number of GST pseudogenes within a species ranged from 4 to 24 and only Alpha and Mu pseudogenes were found ubiquitously in all 22 mammals. Dog had the most GST pseudogenes and 62.5% of its pseudogenes belonged to the Pi class.

**Fig 2:**
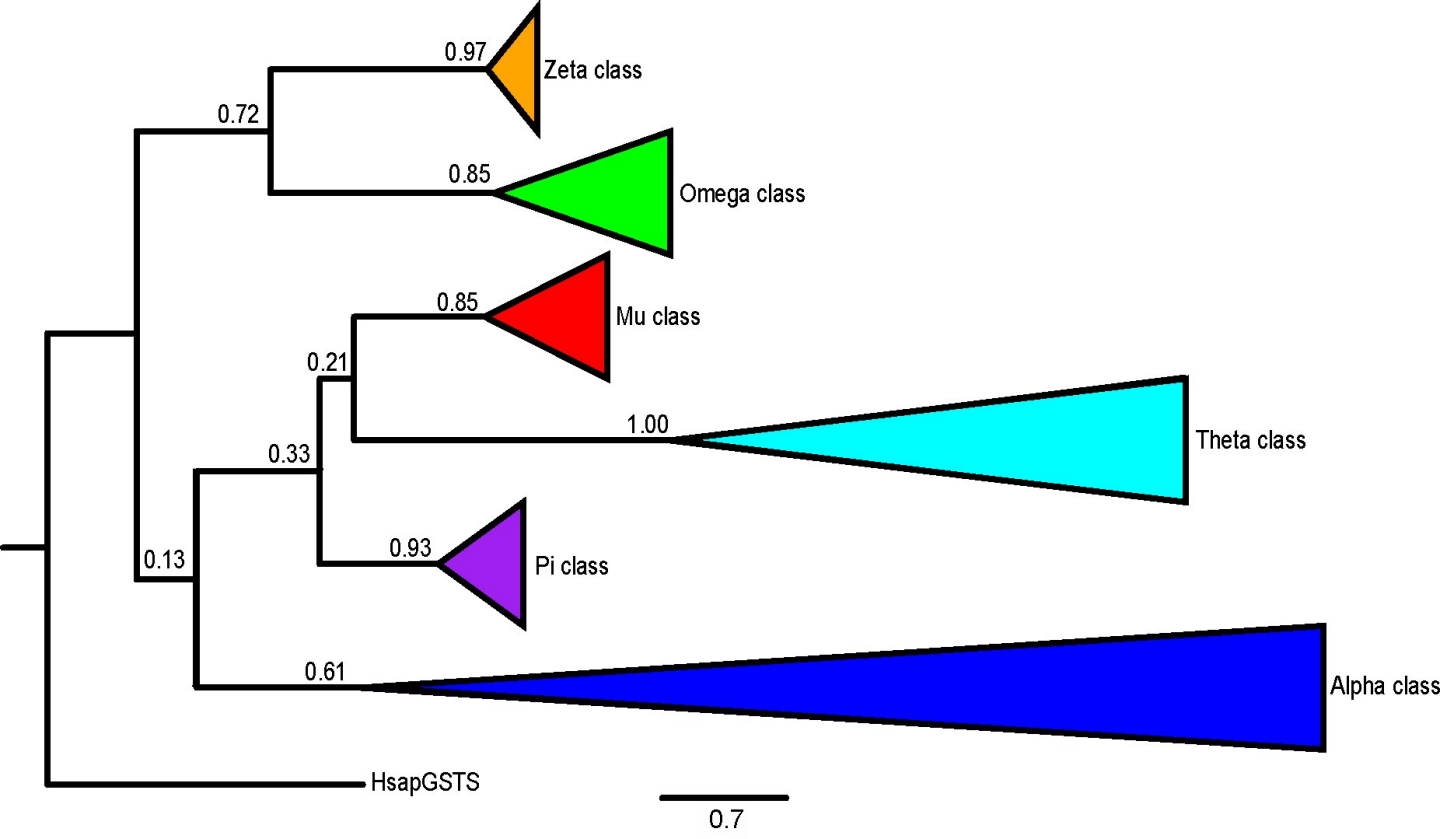
Maximum likelihood tree with 100 bootstraps. Phylogenetic tree of 383 GSTs genes were collapsed into a clade within a class. The human GSTs Sigma (PTGES) was used as an outgroup. Bootstrap values are shown at the internal node.

In order to determine orthologous or paralogous relationships, a maximum likelihood tree was constructed based on the annotated 383 GSTs by using MEGA v6 (Fig 2) [16]. The human GSTs Sigma (PTGES/GSTS) was used as an outgroup and 100-replicate bootstrapped tree was performed. The sigma class GST was an appropriate outgroup since it was not considered as a bona fide GST [9]. With the exception of the Alpha class GSTs, all other classes could be collapsed as nodes with more than 85% bootstrap support. In the assignment of orthologous GST gene set, both the conservation of synteny and the relative position and orientation of genes in the cluster were considered together with nodes that have at least 70% bootstrap support.

### Patterns of mammalian GSTs gene gains and losses

**Fig 3:**
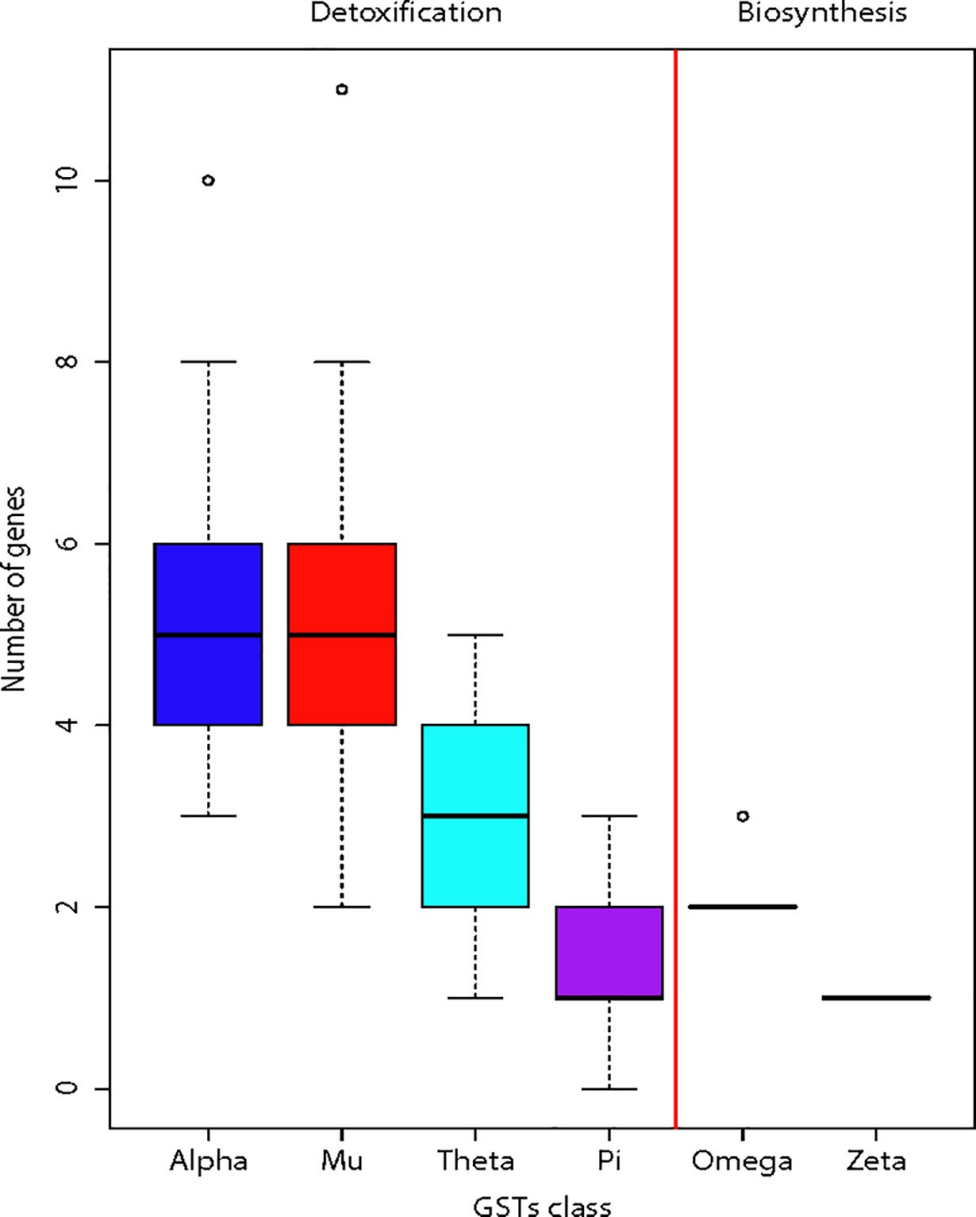
Boxplots showing variation in gene number of each of the six GST classes. From literature searches, the detoxification-type of GSTs are Alpha, Mu, Pi and Theta whereas the biosynthesis-type of GSTs are Omega and Zeta. The red vertical line is used to divide GSTs classes into two different types. Outliers are represented as circles.

**Fig 4:**
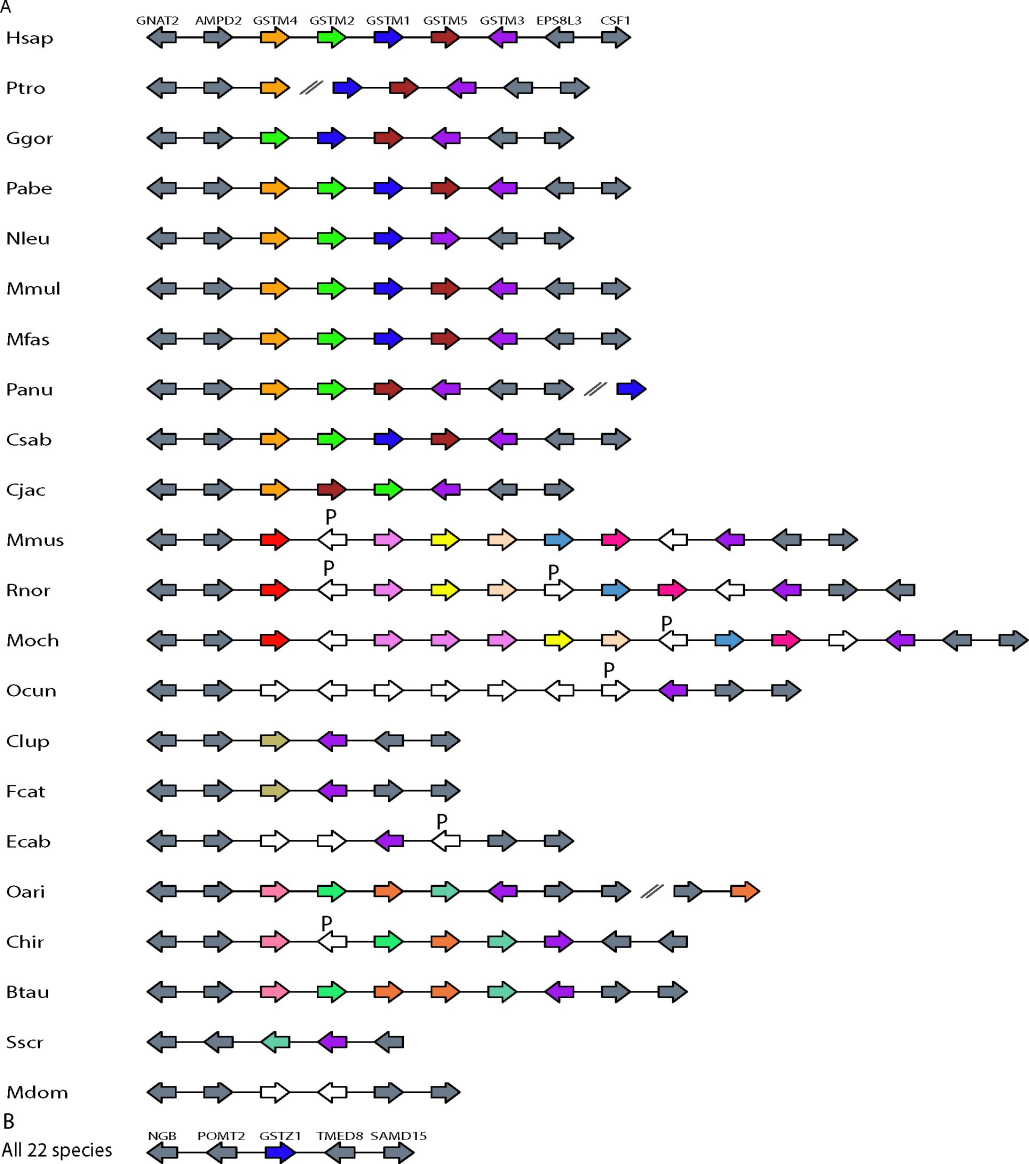
**Gene synteny of GSTs (A) Mu and (B) Zeta of 22 mammalian species.** GSTs and their neighbour genes were indicated as arrow boxes with the arrow head pointing in the direction of transcription. GST gene was labeled as "GST" (e.g. GSTM1). Pseudogene was labeled by "P". The ortholog and paralog set were coloured based on bootstrap value ≥ 70. For those GSTs that lacked bootstrap support but could be grouped as orthologs based on gene order and conserved flanking gene, they were also coloured. Neighbour non-GST genes were coloured grey. Pseudogene and GST gene that were not supported by bootstrap value and gene order are white in colour. The contig break is represented by the "//" symbol.

GSTs could be categorised as detoxification- or biosynthesis-type from literature searches on known functions of these proteins (S2 Table). The number of detoxification-type GSTs (Alpha, Mu, Pi, Theta) across the 22 species showed more variation when compared to the biosynthesis-type (Omega, Zeta) (Fig 3). Among the detoxification-type GSTs, the GST Mu class showed the most variation and its gene cluster reveals complexity in evolution with pseudogenes still present in the cluster in some species (Fig 4A). Additionally, no Mu GST showed clear orthologous relationship that extended from human to opossum. This could be a sign of more rapid evolution in detoxification-type GST, which obscured orthologous relationships. In contrast, the biosynthesis-type GSTs such as the Zeta class showed the least variation in GST gene number across the 22 mammalian species (Fig 4B). In each species, the gene order of Zeta GST and the two flanking non-GST genes were completely conserved.

**Fig 5:**
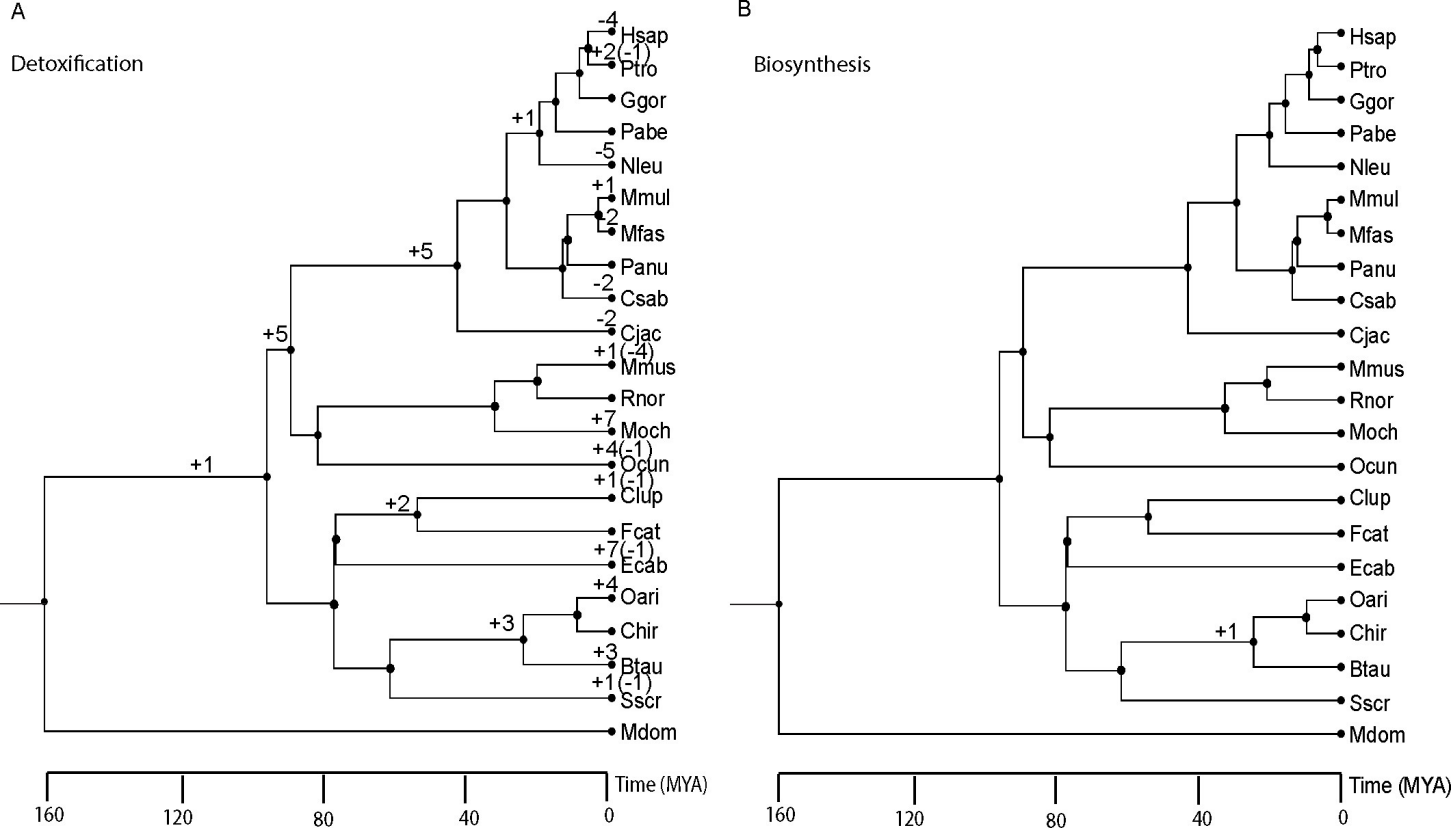
**Pattern of gene gain and loss in (A) detoxification and (B) biosynthesis-type of GSTs.** The “+” and “-” signs indicate the number gene gain and gene loss obtained under the BDIE model, respectively. The tree was obtained from TimeTree.

In order to compare the rates of gene gains and losses of GSTs from detoxification-type (i.e. Alpha, Mu, Pi, and Theta) with biosynthesis-type (i.e. Omega, Zeta), the total number of gene gains and losses events were estimated using DupliPHY-ML [21] parsimony, BDI and BDIE models (Fig 5, Table 1, S5 Table). Pseudogenes were excluded in the analysis. The parsimony model allows gene gain and gene loss to have a constant probability in birth, death and innovation parameters. The BDI model allows estimation of the rate of gene gain (B), gene loss (D), and innovation (I) parameters using maximum likelihood method. The BDIE model added extinction (E) parameter to the BDI model to take into account loss of gene family. DupliPHY-ML gave estimates of sizes of each GST class per branch along the 22 species phylogenetic tree. For interpretation of results, we favoured the BDIE model because it achieved the highest likelihood score (-159.132) among all models.

**Table 1:** Number of gene gains and losses for six GST classes using the BDIE model.

Class	Gene gain	Gene loss	Pseudogene^a^	Type	Total^b^	Total by GST type	Weighted^c^
Alpha	17	13	50	Detoxfication	30		
Mu	17	3	47	Detoxfication	20		
Pi	8	1	22	Detoxfication	9		
Theta	6	7	16	Detoxfication	13	72	22
Omega	1	0	0	Biosynthesis	1	1	1
Zeta	0	0	0	Biosynthesis	0		

^a^The number of pseudogenes only includes those with stop codons as the inactivating mutations.

^b^The “Total” column is the sum of only gene gain and loss events excluding the pseudogene counts.

^c^The “Weighted” column is the result of scaling detoxification-type of GST by division with 3.3 to make it comparable to the biosynthesis-type since it has 3.3 times more ancestral copies.

According to the BDIE model, the ancestor of the 22 mammalian species chosen had 10 detoxification-type (5 Alpha, 2 Mu, 1 Pi and 2 Theta) and 3 biosynthesis-type (2 Omega, 1 Zeta) of GSTs. More ancestral genes should equal to more chances of having gene gains and losses. Therefore, the 72 gene gains and losses events found in detoxification were scaled by division with 10/3 to give 22 events and hence allowed for a fairer comparison given that it had more ancestral genes than the biosynthesis-type. After adjustment for ancestral genes, there were 22 gene gains or losses in detoxification-type GSTs whereas the biosynthetic-type only had one such event. To further quantify the relative increase in rates of gene gains or losses in detoxification-GSTs, we considered the probability of such molecular events in our observed data by using binomial distribution. Under the assumption that each gene gain or loss event was independent and had equal probability to occur in both detoxification and biosynthetic types (i.e. *P*(*biosynthesis*) = *P*(*detoxification*)), then the probability of finding less than or equal one event for the biosynthetic type was *P*(*biosynthesis* ≤ 1) = 2.86*e*^−6^. The probability of gene gain or loss event in detoxification was at least four times higher than the biosynthesis-type (P-value = 0.0398), which supported the rapid birth-death evolution mode in genes involved in detoxification.

### Identification of new positively selected sites

Evidence of adaptive evolution can be detected in a few ways and one method is through identification of higher rates of nonsynonymous to synonymous substitutions. The availability of many sequenced mammalian genomes diverged at different levels allowed for identification of sets of GSTs for searches of positively selected sites. The CODEML tool used here is powerful enough that specific codon under positive selection could be identified. To be specific, we used likelihood ratio tests (Model M8 over M7) to detect positive selection. Of the 21 orthologous sets of GSTs used for searching positively selected sites, only two GSTs (GSTA3 and GSTO1) were found significant after correction for multiple tests using False Discovery Rate P-value less than 0.05 (Tables 2 and 3). A total of six positively selected sites have been identified. One of them was 128G in GSTO1 (P-value = 0.00044). The remaining five positively selected sites were found in GSTA3 (P-value = 1.5×10^−8^) and two of the sites were a new discovery.

**Table 2:** Site models of CODEML for GSTA3 and positively selected sites.

Model	Log-likelihood	2Δ(In L)	P-value^b^	Positively selected sites^a^	Tree length	Average dN	Average dS
M0	-7209.24						
M7	-6956.95						
M8	-6938.93	36.03	1.5×10^−8^	6V, 108Y, 112M, 208D, 221S	7.17	0.0296	0.0700

^a^Bayes Empirical Bayes (BEB) was used to calculate posterior probabilities and only those with *Prob*(*ω* > 1) > 0.95 are shown. Amino acid position follows *Mus musculus* GSTA3.

^b^False discovery rate P-value was only calculated for the M8 vs M7 model.

**Table 3:** Site models of CODEML for GSTO1 and positively selected sites.

Model	Log-likelihood	2Δ(In L)	P-value^b^	Positively selected sites^a^	Tree length	Average dN	Average dS
M0	-4360.09						
M7	-4233.79						
M8	-4226.06	15.46	0.00044	128G	3.74	0.0227	0.0598

^a^Bayes Empirical Bayes (BEB) was used to calculate posterior probabilities and only those with *Prob*(*ω* > 1) > 0.95 are shown. Amino acid position follows *Homo sapiens* GSTO1.

^b^False discovery rate P-value was only calculated for the M8 vs M7 model.

## Discussion

Gene members of multigene families are known to be phylogenetically unstable and the literature is replete with such examples in the immune system (e.g. immunoglobulin [29], major histocompatibility complex [30], T-cell receptor [31]), sensing (e.g. olfactory receptor [32, 33]), chemosensory [34], and others. In the context of detoxification, the phase I cytochrome P450s have gene members that could be grouped by functions into two categories, which are detoxification and biosynthesis [4, 5]. Comparison of gene gain and loss in the cytochrome P450s showed those that are involved in detoxification displayed rapid birth-death evolution. Moreover, the genes involved in detoxification also showed higher rates of evolution [5]. The current work extended previous knowledge on the evolution of multigene families to the phase II GSTs, which is a multigene family generally known for its role in detoxification. Similar to the p450s, GSTs also have gene members that have biosynthesis roles. The finding that GSTs also showed rapid birth-death evolution for the detoxification-type suggests that the enzymes from phase I may have acted in concert with phase II enzymes during evolution.

Recently, the gene family arylamine N-acetyltransferases (NATs), which is another phase II enzyme has been shown to be phylogenetically unstable too [7]. However, the previous study was unable to compare detoxification versus biosynthesis-type because all NATs are thought to function only in detoxification. In addition, NATs are only found as two gene copies in humans and hence, the study of the evolution of this multigene family would not encapsulate the complexity seen in the cytochrome P450s or the GSTs. Nonetheless, the findings from cytochrome P450s, GSTs and NATs support the idea that detoxification genes generally have birth-death evolution mode. This has broad implication on our understanding of how detoxification system has evolved and it remains to be seen whether phase III genes also displayed the same evolution pattern.

It was interesting to observe that dog has the most GSTs pseudogenes and 15 out of 24 of its pseudogenes belong to the Pi class. Of the 15 Pi pseudogenes, 11 of them appear to be processed pseudogenes created by reverse transcription of processed RNAs and subsequent genomic integration of the cDNA. These processed pseudogenes lack introns and have poly-A tail. It was thought that processed pseudogenes must be expressed in the germline to facilitate their fixation in the genome early in development and to be inherited [35]. Until recently, it was not known what might explain the high number of processed pseudogenes. Studies in glycolytic genes have shown that those genes with a high level of gene expression is correlated with having more processed pseudogenes [36]. There is also a dependency on Long Interspersed Nuclear Element (LINE) activity since the reverse transcriptase produced by them is what drives the retrotransposition of other genes. To our knowledge, relative gene expression of GST Pi in dog has not been studied in detail in the germline and the relationship of LINE activity with GSTs have not been explored. Our results suggest GST Pi is likely highly expressed in the germline, which coincided with LINE activity in the past and led to fixation of many processed pseudogenes observed in the genome.

The evolution of GSTs in response to changes of environmental toxins and niche-defining feeding substrates makes them excellent targets to study adaptive evolution. Indeed, many studies have found evidence of amino acid sites in GSTs under positive selection [37] and some studies have even tested the potential functional significance of the residues using *in vitro* biochemical study [10]. For example, the residue 210 in GSTM2 has a biochemically conservative threonine conversion to serine but it showed a thousand-fold increase in substrate specificity when mutated.

Determination of positively selected sites relies on the accuracy of gene annotation [38]. Surveys of positively selected sites that placed confidence on gene models solely from the NCBI automated genome annotation pipeline may result in both false positives and false negatives. The current study took a painstaking approach to curate gene models prior to using them to search for positive selection. Given the differences in our gene models and chosen species, our results were not the same as previous positive selection screens on the GSTs [10, 37]. Additionally the positive selection screen was done by ensuring there were sufficient power to detect selection by using adequate tree branch lengths and at least 6 taxa [28]. The problem of multiple mutations occurring at the same site was also ruled out by ensuring both average dN and dS are less than 1. Furthermore, correction for multiple tests was applied to avoid inflation in chances of getting a significant P-value.

The finding of positively selected sites 128G in GSTO1 and 112I, 208E in GSTA3 that agreed with the literature [37] suggested our screen was dependable. Inspection of human 3D structure (PDB accession: 1eem) showed 128G in GSTO1 is located opposite to GSH at a distance of 10.3 Å, which due to its close proximity to the active site, it may play a role in substrate binding. GSTO1 could have been selected for detoxification purpose given that it has better activity against arsenic but performed poorer in ascorbate metabolism than GSTO2 [37, 39].

Mouse GSTA3 is responsible for aflatoxin resistance and evidence for this trait was shown by aflatoxin B_1_-8,9-epoxide (AFBO) sensitivity in GSTA3 (-/-) knockout mice [40] and the high AFBO catalytic activity in GSTA3 [41]. Alfatoxins are a family of toxins formed by the common fungal molds *Aspergillus flavus* and *Aspergillus parasiticus*, which tend to be abundant in warm and humid environment. Both human and primates do not have clear orthologs to mouse GSTA3 and none of their GSTs show high catalytic activity against AFBO. Amino acid residues important for GSTA3 aflatoxin detoxification were identified in a site-directed mutagenesis study coupled with *in vitro* biochemical testing for activity against AFBO [41]. Since rat GSTA3 has 86% identity with mouse GSTA3 but showed almost no detectable activity against AFBO, its protein was mutated to identify residues that conferred AFBO activity. No single amino acid residue elevated rat GSTA3 AFBO activity with the exception of E208D substitution that only marginally increased activity. When a series of amino acids were mutated based on molecular modelling that indicated these residues to be near or in the active site, it was found that six residues (E104I, H108Y, Y111H, L207F, E208D, V217K) elevated the rat GSTA3 AFBO activity to 40 nmol/mg/min from a baseline of negligible activity. However, the best combination of mutated amino acids in rat GSTA3 was still ~6.6 times less than the wild-type mouse GSTA3 AFBO activity, which suggested some critical residues important for the aflatoxin detoxification was still unknown. Two of the residues, 108Y and 208D, in the mutant protein with the highest AFBO activity are positively selected sites in this study. The functional significance of the other three positively selected residues (6V, 112M, 221S) warrant further investigation as all five residues may play a role in aflatoxin detoxification.

## Conclusion

The culmination of molecular evolutionary analyses on multigene families with members playing a role in detoxification such as cytochrome P450s, arylamine N-acetyltransferases and GSTs all lead to birth-death evolution mode and positive selection as hallmarks of detoxification genes.

## Supporting information

S1 Table Annotation details of GST genes, pseudogenes, partial genes and curated genes across the 22 mammalian species. (XLSX)

S2 Table Classifications of each GST class as generally detoxification or biosynthesis-type from literature survey. (XLSX)

S3 Table A list of known positively selected sites in mammalian GSTs from literature searches. (XLSX)

S4 Table Metadata of genome assemblies of the 23 chosen mammalian species. (XLSX)

S5 Table Inference of GSTs gene gain and loss using Pasimony, BDI and BDIE models in DupliPHY-ML. (XLSX)

## Abbreviations

GST: Glutathione S-Transferases
NCBI: National Center for Biotechnology Information
PDB: Protein Data Bank
NAT: arylamine N-acetyltransferases
